# Valorization of *Posidonia oceanica* balls: sustainable simultaneous synthesis of green de-icer and eco-friendly adsorbent for saline water treatment from methylene blue

**DOI:** 10.1038/s41598-026-66019-w

**Published:** 2026-08-13

**Authors:** Khaled S. Abou-El-Sherbini, Randa R. Elmorsi

**Affiliations:** 1 Department of Inorganic Chemistry, National Research Centre, 33 El Bohouth st. (former Eltahrir St.), P.O. 12622, Dokki, Giza Egypt; 2 National Institute of Oceanography and Fisheries (NIOF), Cairo, Egypt

**Keywords:** Neptune balls, Biomass valorization, Methylene blue, Sustainable adsorption, Green deicer, Calcium magnesium acetate, Chemistry, Environmental sciences, Materials science

## Abstract

Neptune beach balls (NB) are natural waste materials often found along the Mediterranean Sea coasts. NB are originated from the decay of *Posidonia oceanica* (PO) organs leaving balls of the plant fibers, sand and impurities. Due to upcoming global warming, the accumulation of contaminants-loaded NB among PO wastes, they are expected to intensify, increasing the environmental challenges posed by these eco-wastes. This study presents a novelle sustainable acetic-acid activation strategy for the double valorization of Neptune balls (NB) into an eco-friendly adsorbent and green deicer. The materials were characterized using ICP-OES, FTIR, Raman spectroscopy, XRD, TGA, SEM-EDX, zeta potential, and/or pH_PZC_ analyses. Through a simple and economical treatment process, 84.5% of the cleaned NB waste is transformed into a natural adsorbent. Simultaneously, the circular economy is approached by valorizing the acid activation liquor waste into an environmentally friendly calcium magnesium acetate (CMA) deicer that was recovered with approximately 83% efficiency, as a by-product adding value to the NB valorization process. The natural adsorbent was capable of removing up to 97.5% of MB from freshwater and saline water, with a monolayer MB adsorption capacity of 135 mg g^− 1^. The MB adsorption is well fitted by the Langmuir and Freundlich models while following the pseudo-second-order kinetics model. MB-multilayer was suggested to be adsorbed primarily by electrostatic attraction. The activated NB has been successfully tested for the removal of MB dye from seawater spiked with MB, while CMA demonstrated better deicing performance compared to conventional sodium chloride salt, highlighting the potential to advance the economic valorization of NB waste, supporting both water treatment and eco-waste management.

**Supplementary Information:**

The online version contains supplementary material available at 10.1038/s41598-026-66019-w.

## Introduction

*Posidonia oceanica* belongs to the *Posidoniaceae* family. It is an effective ecosystem for cleaning seawater from toxic metals by absorbing them from the Mediterranean Sea, which receives millions of tons of harmful waste annually^1–5^.

Neptune balls (NB), also known as *P. oceanica* balls, are common natural waste found along Mediterranean coasts. These fibrous spherical formations result from the wave-induced abrasion and tangling of shed *P. oceanica* fibers, which naturally detach due to sea currents and constitute approximately 30% of *P. oceanica* litter^6,7^. Although NB formation is a natural phenomenon, their accumulation poses environmental challenges, such as impacting coastal aesthetics and potentially disrupting local ecosystems, which in turn affects tourism, a crucial economic sector for many Mediterranean countries^8–10^. Additionally, unmanaged waste can contribute to the degradation of coastal ecosystems. Traditional disposal methods, such as burning or landfill deposition, can lead to further environmental issues, including air pollution and soil contamination- particularly due to the harmful residues they contain such as trapped plastics^1,11^.

Valorizing this waste not only provides environmental benefits but also presents significant economic opportunities^10,12,13^. In recent years, research has increasingly focused on sustainable methods for managing and utilizing this waste^10,14^. Restaino et al. (2023) emphasized the potential of NB as a sustainable resource, emphasizing the need for innovative approaches to transform environmental challenges into economic assets^14^. NB represent a biomass of plant lignocellulose that can serve as a sustainable substrate for producing value-added molecules in technological bioprocesses, as bio-adsorbents in environmental cleansing, for the preparation of bioplastics and new bio-composites, or as insulating and reinforcing materials in construction^14^. Carbohydrates account for 28.3% of the NB dry mass while 72.5% of the NB dry mass is lignin^12^ while Rencoret et al.^15^ stated that NB contain the highest lignin amount (44%) compared to other PO parts. Eight monosaccharides have been identified, with glucuronic acid (46.6%) and rhamnose (29.6%) being the most prevalent in the lignin. Thus, the extracted lignin was defined as a water-soluble carbohydrate / lignin compound consisting of phenolic polymer chains covalently linked to hemicellulose fragments, exhibiting remarkable antioxidant activity^12^. Lignin was separated, and cellulose nanofibers were obtained through chemical and mechanical treatment^10^. Pfeifer (2021) suggested that NB fibers could be used as enhanced polymer additives, finding xylan to be the most abundant saccharide, which can be reused in biofuel and bioplastic production^7^. Fulignati et al.^16^ reported the valorization of NB for extracting organic extractives and lignins and producing butyl levulinate via a series of expensive and tedious extraction and hydrolysis processes.

The economic exploitation of NB holds significant potential. One promising avenue is the production of natural adsorbents from treated NB. Adsorbents play a crucial role in water purification, particularly in the elimination of hazardous cationic dyes from industrial effluent. The worldwide mandate for effective adsorbents is increasing, and the development of cost-effective natural alternatives can reduce reliance on synthetic products, thereby lowering production costs and environmental impact.

The discharge of wastewater containing dyes from industrial activities into natural water bodies is an increasing concern due to its negative effects on aquatic life. For instance, in the textile industry, manufacturing one kilogram of the final product consumes between 150 and 200 L of water, with 125 L becoming polluted water^17^. Consequently, the daily discharge of hazardous contaminated water is very high in these industries. Heavy metal ions as well as countless dyes are non-biodegradable and lead to severe healthiness impacts even at small concentrations. Therefore, efficient and economical wastewater treatment is important to diminish contaminant concentrations to tolerable levels before discharge into water sources.

Calcium and magnesium acetate are economically required as an environmentally friendly ice solvent which has not been previously prepared from PO. However, CMA was reported to be synthesized from dolomite^18,19^ or corn cob ash^20,21^. Calcium and magnesium acetate was also utilized as a highly efficient energy store^22^.

Although NB was previously demonstrated promising heavy metal adsorption capacities ranging from 233.3 to 698.9 µmol g^− 1^, it remains unclear whether this adsorption capacity is attributable to the fibrous matrix itself or to deposited contaminats^23^. In contrast, PO leaves and rhizomes have been successfully activated and applied for saline-water remediation; however, to the best of our knowledge, the activation and utilization of NB have not yet been investigated, although it constitutes approximately 30% of PO beach-cast wastes^24,25^.

The present study addresses these knowledge gaps by developing a simple and low-cost and environmentally friendly approach for the valorization of Neptune ball with potential economic benefits. A mild acetic-acid activation was employed, combined with physicochemical characterization including FTIR, Raman spectroscopy, XRD, SEM-EDX, TGA, zeta potential, and pH_PZC_ analyses to evaluate the suitability of activated NB for saline-water remediation from methylene blue as an example of cationic dyes.

In addition to producing a value-added eco-adsorbent, the proposed process enables the recovery of economical useful compounds from NB. In particular, acetate based recovered during processing can serve as environmentally friendly de-icing agents, which are widely used to maintain safe transportation in cold regions. By integration cost-effective waste utilization with resource recovery, the proposed approach overcomes the limitations associated with previous studies and offer both environmental sustainability and economic feasibility, transforming Neptune beach ball waste from an environmental nuisance into a valuable resource.

The novelty of this work lies in the first reported simultaneous dual valorization of Neptune balls into both a high-performance bioadsorbent and a green de-icing product. Additional innovative aspects include the use of a mild and environmentally benign acetic acid activation process, the evaluation of adsorption performance under saline-water conditions, the assessment of the antibacterial properties of the activated material, and the integration of circular-economy principles through comprehensive biomass valorization.

## Experimental

### Materials

Glacial acetic acid was commercially procured from Domar & Sons (Adra, Syria). Methylene blue (MB, C_16_H_18_ClN_3_S·xH_2_O > 96.0%) was bought from S D Fine-Chem Limited (Mumbai, India). Stock solutions (1000 mg/L) were set by dissolving one gram of MB in one liter of distilled water (DW). Additional substances and chemicals were of Puriss grade from Sigma-Aldrich unless otherwise specified.

Neptune beach ball waste was collected from the Ajami beach at kilometer 21, west of Alexandria, Egypt, location 31.102621046423586, 29.741497168319086. For practical application studies, surface water samples were obtained from various sites along the Egyptian Red Sea coast at Gouna Suez, representing different salinity levels based on the water flow.

### Equipment

Elemental analysis via inductively coupled plasma optical emission spectrometry (ICP-OES) was performed using an iCAP-7400 ICP spectrometer (Thermo-Fisher Scientific, Germany) to determine the concentrations of elements such as Li, K, Mg, Ca, Sr, Ba, B, Al, Cr, Mn, Fe, Co, Cu, Zn, and Cd in digested samples of NB. Samples were digested in a MILE STONE CONNECT ETHOS EASY microwave oven using 0.5 g of each sample in eight mL of concentrated HNO_3_ and two mL of concentrated H_2_O_2_. Single-element standard solutions (Fisher Scientific) were used to make multi-element standard solutions at required concentrations, which were then used to establish the calibration curve.

Attenuated Total Reflectance - Fourier Transform Infrared (ATR-FTIR) spectra of Neptune ball samples were obtained on a Bruker ALPHA-FTIR-Spectrometer with a platinum attenuated total reflectance (ATR) module, covering a range from 400 to 4000 cm^− 1^. Fourier transform infrared (FTIR) spectra of calcium magnesium acetate samples were recorded using a Nicolet iS10, Thermo-Fisher Scientific, USA, using KBr pellet - technique. Total nitrogen, carbon, hydrogen, and sulfur in the samples were measured using a Thermo Scientific Flash2000 NC analyzer (Italy), model: Flash2000, serial number: 2015F0028.

X-ray diffraction (XRD) measurements were conducted using a Bruker AXS D8 ADVANCE instrument with Cu K_α_ radiation (λ = 1.5406Å) operated at 40 kV and 40 mA. Thermogravimetric analysis (TGA) was done using an SDT Q600 (V20.9 Build 20) instrument with alumina crucibles, from room temperature to 1000 °C at a rate of heating of 10 °C/min under N_2_.

Raman shift spectra, the percentage of C, H, N, and S, the point of zero charge (pH_PZC_) of the adsorbents, UV/Vis spectra and Colony Forming Unit (CFU) Test were performed as detailed in S1 (supplementary information).

Chemical analyses of major oxides (wt%) and some trace elements in the de-icing agent were performed using an X-ray fluorescence (XRF) spectrometer (Axios, WD-XRF Spectrometer, PANalytical, 2005, Netherlands).

UV/Vis spectra of MB solutions were recorded using a UV/VIS Peak Instrument – C7200 Spectrophotometer (PRC) at 663.8 nm. The concentration of MB was determined using a calibration curve prepared from standard MB solutions. The zeta potential of the aqueous sample suspensions was measured using a Zeta Sizer, ZS-Nano, from Malvern Instruments Ltd. (Malvern, UK). For zeta potential measurements, the sample was sonicated for 30–60 min before evaluation.

The initial pH of sample solutions was measured with an ADWAA AD8000 pH/TDS/mV meter (Romania), which has an extended range and an accuracy of ± 0.01 pH. The same device was used to measure the pH and total dissolved solids (TDS ± 0.1 ppt) in seawater samples.

### Methodology

#### Preparation of NB for processing

NB (supplementary information, Fig. S2) were sorted, washed thoroughly with tap water and skimmed to remove incorporated sand, then washed using distilled water and dried at 70 °C. They were sorted again, cut, and the fibers were sieved through a mesh with a one mm pore diameter. The resultant material, representing 75% of the initial waste, was termed washed NB (NBW).

### Acid-treatment of NB

For single acid treatment, NBW was soaked in a 2.5% acetic acid solution (mass/volume ratio of 1:10) overnight, then skimmed, washed carefully with distilled water. The dried extract was recrystallized, resulting in crude calcium and magnesium acetate deicer, which was recrystallized, termed CMA1 and amounted 11.5% (supplementary information, Fig. S3). The acid-activated NB were then boiled in distilled water for half an hour, washed with distilled water, dried at 70 °C for 24 h. The treated NB, representing approximately 89%, were stored in polyethylene bags and termed ANB1.

For triple-acid-treatment the untreated Neptune ball fibers NBW were soaked in a 2.5% acetic acid solution (mass/volume ratio of 1:10) overnight, then skimmed, and the soaking process was repeated twice using 0.25% acetic acid. The extracts were collected, filtered, concentrated, dried at 70 °C, and recrystallized to yield crude calcium and magnesium acetate deicer, which were recrystallized, amounted 14.0% and termed CMA2 (supporting information, Fig. S4). The acid-activated NB were then boiled in distilled water for half an hour, washed and dried as above mentioned. The triple-acid-treated NB, representing approximately 84.5%, were stored in polyethylene bags and termed ANB3 (supplementary information, Fig. S5).

#### Adsorption studies

Optimization experiments were conducted in triplicate and reported as mean ± standard deviation. Adsorption isotherm and kinetic profile measurements were performed as single measurements after confirming reproducibility during preliminary optimization experiments and observing consistent adsorption trends without abnormal deviations.

Adsorption kinetic and equilibrium isotherm experiments were conducted as representative single measurements after preliminary optimization experiments had confirmed good reproducibility and consistent adsorption behavior. The detailed adsorption procedures are provided in Supplementary Information (Section S6).

### Applications

#### Treatment of MB-spiked seawater with activated NB

A batch experiment was conducted for the adsorption of MB from natural water samples spiked with 50 mg L^− 1^ MB. An ANB3 dose of 1.0 and 2.0 g L^− 1^ was shaken in 5 ml of water sample for 60 min at ambient pH and room temperature. The samples were then centrifuged and the initial (C_i_) and final (C_f_) MB concentrations were determined. The removal efficiency (E_R_%) was calculated using the following formula:

1 $$\:E_{R} = \frac{{C_{i} - C_{f} }}{{C_{i} }} \times 100\%$$

#### Deicing application with CMA1

One, two, or three grams of CMA1 deicer were sprinkled on 20 g of ice. For comparison, a similar experiment was conducted under the same conditions using fine sodium chloride instead of CMA1. The weight of the melted portion of the ice was monitored every 20 min. The deicing experiment was performed once as a proof-of-concept demonstration.

### Statistical analysis

Optimization experiments (initial MB concentration of 100 mg/L, adsorbent dose of 2.0 g/L, shaking time 60 min, initial pH values of 5.8 and 11.2) were performed in triplicate (*n* = 3), and the results are expressed as mean ± standard deviation (SD). Error bars shown in the corresponding figure represent ± SD.

For error bar calculations, standard deviation (SD) is calculated according to the following equation:

2 $$\:SD = \sqrt {\frac{{\sum {\left( {E_{{R,i}} - \bar{E}_{R} } \right)^{2} } }}{{n - 1}}}$$

Where, $\:\genfrac{}{}{0pt}{}{-}{{E}_{R}}$ is the mean removal efficiency, $\:{{E}_{R}}_{,i}$ is removal efficiency obtained in the *i*th replicate, and n is the number of replicate measurements. Error bars represent ± SD based on the replicate experiments.

Adsorption kinetic and equilibrium isotherm experiments were conducted as representative single measurements after preliminary optimization experiments had confirmed good reproducibility and consistent adsorption behavior.

The seawater application experiments were performed once for each sampling site as practical demonstration experiments using five independent seawater samples collected from different locations.

The deicing experiment was conducted once as a proof-of-concept comparison between calcium magnesium acetate and sodium chloride.

Pearson’s two-tailed bivariate correlation analysis was performed using IBM SPSS Statistics software to evaluate the relationships between water quality parameters (pH and TDS) and methylene blue removal efficiency (E_R_). The analysis was used to determine the strength and significance of the linear associations among the studied variables. Statistical significance was accepted at *p* < 0.05^26,27^.

## Results and discussion

### Characterization of NB

The analysis of some metallic and metalloid elements in untreated NB (NBW) is shown in Table 1. The results indicate the presence of metal and metalloid elements up to 3.11%, of which calcium being the highest, followed by magnesium, boron, iron, sodium, potassium, and aluminum. When comparing calcium, magnesium, and boron contents in the NB’s, which are the most soluble components in acetic acid, their concentrations decreased from 20,015, 4776, and 2835 mg/kg in NBW to 3901, 308, and 229 mg/kg in ANB3, representing elemental release of 80.5%, 93.6%, and 91.9%, respectively. Meanwhile, there was no significant change in aluminum, chromium, and iron concentrations.

The results shown for Zn contents of NBW are within the reported by Serrano et al.^28^ for PO mat while lower than Al, Ca, Fe ad Mg contents of NBW from Kefalonia Island^29^. The levels of mineral elements in NBW are significantly higher compared to single-acid treatment (ANB1) and triple-acid treatment NB (ANB3), where the levels of these elements are substantially reduced down to only 0.63%, indicating the effectiveness of the treatment procedure and the necessity of the triple-stage treatment. Acetic acid significantly releases elements and compounds, from the NBW, which were accumulated and/or deposited during the plant’s life cycle in the sea and after its death^2,6,30^.

The release of the aforementioned elements is confirmed by the ash content remaining after burning in air for three hours at 600 °C. The ash content was 8.53% for untreated NB (NBW), while the triple-acid treated NB (ANB3) showed only 1.83% ash (Table 2). Notably, the yield of the activated grass in triple-acid treatment reached 84.5% of the initial mass of NBW, whereas in single-acid treatment, the yield was 89.0%. These yields correspond to the observed decrease in metallic and metalloid content after single and triple treatments, considering the hot water wash step, which removes hot water-soluble substances like starch and some organic compounds, which are undesirable when using single-time acidified grass for water treatment as they increase the Chemical Oxygen Demand (CMD) and thus reduce water quality^7,12,31^.

NB showed a significant increase in its carbon content in the triple-acid-treated grass (ANB3) compared to the untreated (NBW), due to the liberation of some elements and inorganic compounds through acid treatment as demonstrated in Table 2. The acid-treated NB did not show any nitrogen or sulfur content, indicating the release of nitrogen during the acetic acid treatment.

**Fig. 1:**
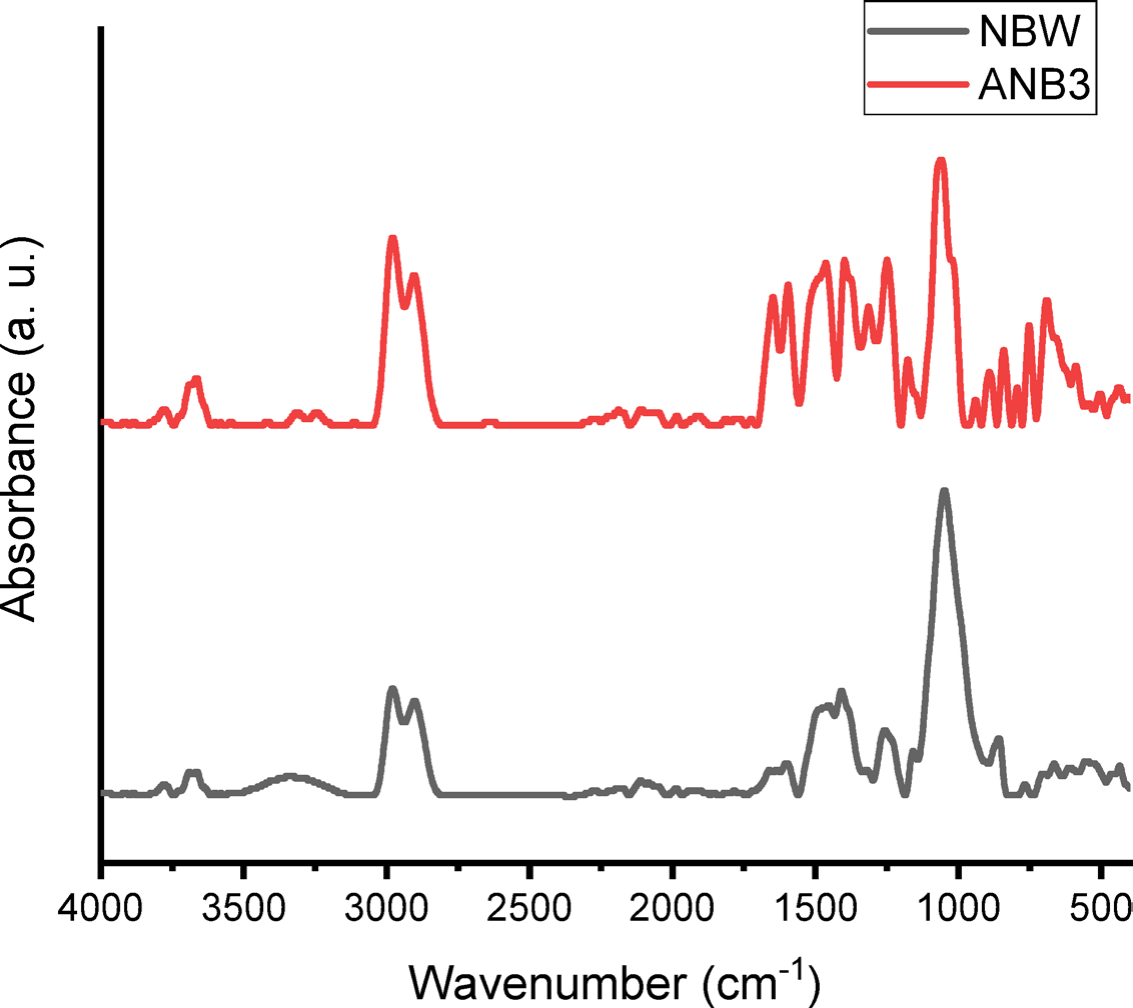
ATR-FTIR spectra of water-washed and acetic acid-treated NB’s.

The FTIR spectra of both untreated NBW and acid-treated (ANB3) (Fig. 1) confirm the effectiveness of acid activation. Several absorption bands were observed in the NBW spectra at 3337, 2988, 2902, 1648, 1599, 1476, 1410, 1251, and 1049 cm^− 1^, which may be corresponding to vibrations of OH groups, symmetric and asymmetric CH_2_, carboxyl groups, aromatic C = C, symmetric and asymmetric C-H bending, C-O stretching in aryl ethers and asymmetric C-O stretching, respectively^25^. The major absorption bands at wavenumbers of 1648, 1599, 1476, and 1251 cm^− 1^ may be attributed to lignin^32^. The spectra of acid-treated grass (ANB3) were more sharp and less overlapped compared with strongest of asymmetric C-O stretching. These variations in addition to the presence of slight shifts may be due to the washing out of deposited carbonates and the freeing of metal-bonded hydroxyls.

**Fig. 2:**
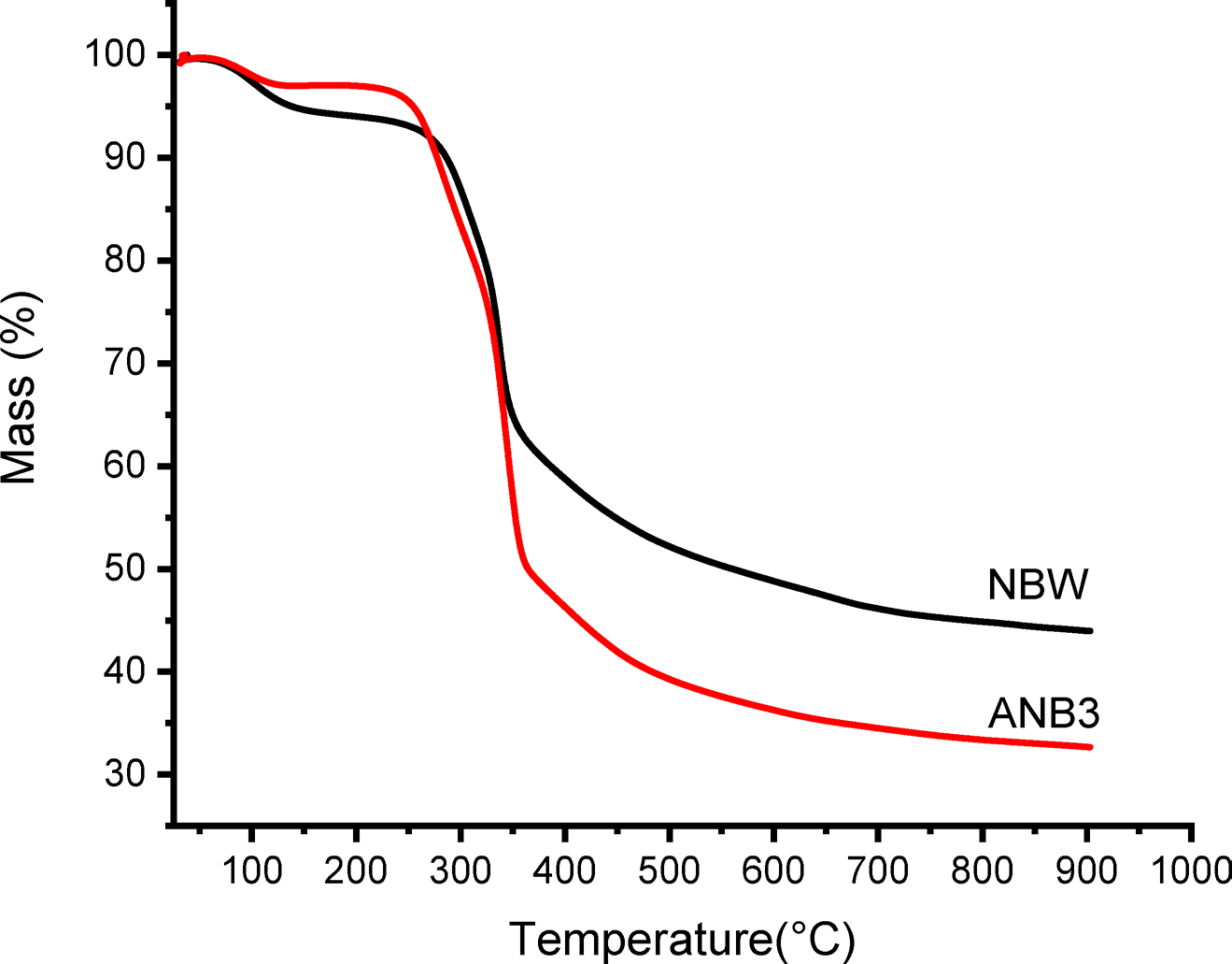
Thermogravimetric analysis (TGA) of water-washed and acid-treated NB.

Figure 2 illustrates the thermogravimetric analysis of NB before (NBW) and after acid treatment (ANB3), showing three main mass loss stages. The first stage, from room temperature to around 140 °C, represents the loss of adsorbed water, accounting for 6.1% and 3.0%, with midpoint temperatures at 102 °C and 95 °C, respectively. The second stage of mass loss began at 283 °C and 252 °C and ended at 345 °C and 355 °C, with mass losses of 33.7% and 48.6%, respectively. It is evident that the second stage in NBW was a single stage at a midpoint of 334 °C, while in ANB3, it comprised two stages at midpoints of 284 °C and 340 °C. The third stage showed a gradual thermal mass loss extending from the end of the second stage to the end of the experiment at 900 °C, with final mass losses of 55.9% and 67.2%, respectively.

These results are in accordance with analytical analysis of the NB samples, which indicate the effectiveness of acid activation, as evidenced by the greater overall mass loss in ANB3 compared to NBW, particularly in the second stage, which represents the loss of most structural water and carbonization^25,33,34^. Petrounias et al.^29^ reported that hemicelluloses/lignin and celluloses/lignin contained in PO biomass show degradation processes, with their maximum located near 260 °C and 334 °C, respectively, which are close to the present results.

Both samples of untreated and treated NB exhibited high negative zeta potentials indicating that they have apparent colloidal stability^35^. The absolute value of zeta potential for NBW (-20.6 mV) is higher than that of ANB3 (-17.9 mV). These values suggest that NBW might have slightly better colloidal stability compared to ANB3, as the stronger negative charge would result in greater repulsion between particles, reducing possible aggregation. Therefore, the acid activation process slightly reduced the negative charge on the particles, as evidenced by the less negative zeta potential of ANB3 compared to NBW. This change could result from metal cation and deposits loss altering the surface chemistry of the particles due to acid treatment, involving the neutralization of some surface charges and the introduction of new functional groups such as protons and hydroxyls instead of carbonate and carboxylate salts, modifying the surface charge^24,25^.

**Table 1:** Elemental analysis: trace and major elements in samples (mg/kg or otherwise stated) compared with reported values.

Element (mg/kg)	NBW	ANB1	ANB3	NBW	NBW	PO rhizomes	PO leaves
Al	451.21	NA	449.56	0.8–1.6%		259.87	300
V	13.31	4.71	5.67			-	-
Ag	23.00	21.80	0.28			-	-
B	2834.9	1243.3	228.78			1924.27	2000
Ba	4.50	5.94	5.91			15.21	–
Ca	20,015	9093	3901	4.2–8.8%		19,172	10,850
Cd	1.08	1.36	0.42			0.65	2.5
Cr	17.04	16.32	17.74			35.60	47.5
Cu	23.34	16.65	15.58			85.44	10
Fe	1031.9	1112.0	925.4	0.4–1.2%		372.49	875
Mg	4776.2	910.6	307.61	0.9–1.1%		7544	4800
Mn	27.19	12.64	6.94			9.39	–
Sr	343.9	125.13	58.81			313.59	–
Zn	47.36	25.80	40.42		23–95	45.95	10
K	635.5	844	149	0.3–0.4%		1560	–
Na	898.0	501.70	159.0	0.8–1.1%		–	–
Total	31,143	13,935	6272			–	
Reference	This work	This work	This work	^29^	^28^	^24^	^25^

**Table 2:** Sample composition: CHNS and Ash Analysis (%) of NBW, ANB1 and ANB3 compared with reported values.

Sample	*N*	C	H	S	Ash%	References
NBW	2.389	43.33	5.4	ND	8.53	This work
ANB3	ND	47.97	5.33	ND	1.83	This work
NBW	-	37.5–44			10.95–11.4	^29^
PO rhizomes	0.29	44.72	4.66	1.65	-	^24^
PO leaves	0.42	37.86	4.93	0.16	12.03	^25^

Table 3 shows the results of CFU count and inhibition percentage of ANB3 against *Staphylococcus aureus*, a model pathogenic bacterium, compared to NBW. The inhibition percentage was 44.2% for the activated NB, while it was only 3.7% for the untreated NB, demonstrating good antibacterial efficacy. This finding aligns with the discovery of bioactive compounds in *P. oceanica* extracts, the primary component of NB [31]. Vasarri et al. also noted its anti-inflammatory properties [36].

In summary, acid-treated NB (ANB3) exhibits significant reduction in metal contents, improved carbonaceous composition, enhanced antibacterial properties and clear visual cleaning effects as shown in their optical microscopic images (supplementary information, Fig. S7), making them promising candidates for water purification and other applications.

**Table 3:** CFU count and inhibition percentage for ANB3 compared to NBW.

Staphylococcus aureus	ANB3	NBW	Control
Dilution Factor	10⁻⁴	10⁻⁴	10⁻⁴
Volume of Broth Plated (µl)	20 µl	20 µl	20 µl
CFU at Dilution Factor	120	207	215
Total CFU/ml	6,000,000	10,350,000	10,750,000
Log Total CFU	6.8	7.02	7.03
Inhibition Percentage	44.2%	3.7%	

### CMA characterization

The percentage of CMA2 product (supporting information, Fig. S4), resulting from the triple acid treatment of the NBW, in respect to the initial NBW, is 14.0%, which is higher than that of CMA1 (11.5%) (supporting information, Fig. S3) resulting from the single acid treatment. This increase in yield applying the triple acid treatment is attributed to the efficiency of removing the metallic content by repeating the acid leaching process three times, as shown in Table 1. Comparing the expected values of metal acetates analyzed in activated Neptune spheres and confirmed by ICP-OES results, they are found to be 9.55% and 13.9%, respectively, showing a remarkable convergence with a slight increase in the actual yield compared to the theoretical values.

This difference is attributed to the exclusion of water of crystallization from the resulting acetates and, to a lesser extent, to the possibility of the presence of elements other than the targeted group analyzed by ICP-OES, as the XRF analysis of CMA1, indicates in Table 4. This analysis shows that calcium and magnesium are the main metallic elements, with impurities of sodium, strontium, silicon, and potassium. The large thermal mass loss is mainly attributed to the organic content of acetate and water of crystallization. The total proportion of acetate corresponding to the mineral content of calcium and magnesium is 72.35% and 22.71%, respectively, or a total of 95.06%, allowing us to disregard the remainder.

**Fig. 3:**
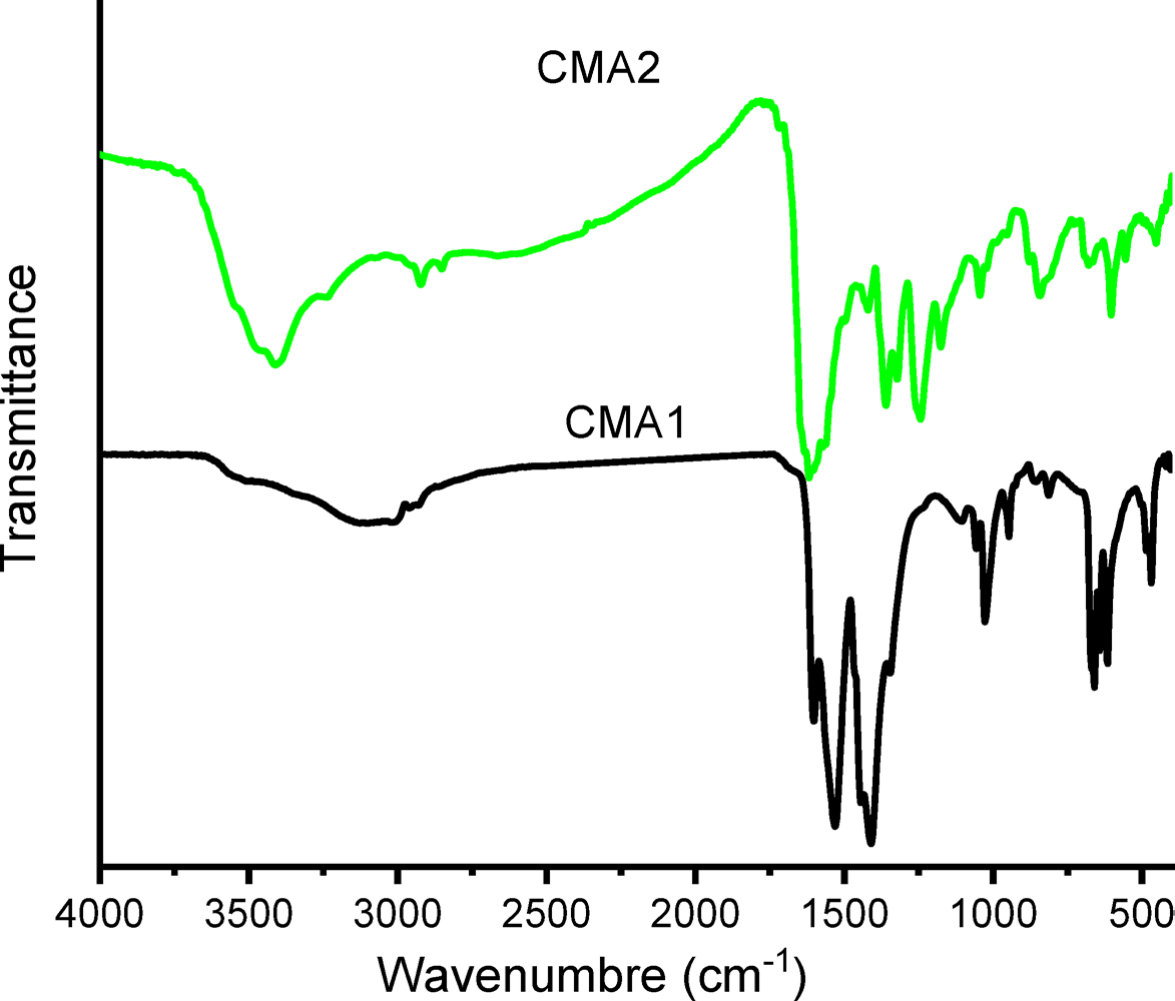
FTIR spectra of raw calcium magnesium acetate de-icing agents.

FTIR of the de-icers CMA1 and CMA2 (Fig. 3) confirms its calcium-magnesium acetate composition. The infrared absorption spectrum of calcium-magnesium acetate, particularly CMA1, matches the typical spectral profile of previously published ice solvents prepared from the combustion of corn cob residues^20^ or from the reaction of dolomite with acetic acid^18,19^. The bands at 3105 (very broad) and 1602 cm^-1^ correspond to the -OH and asymmetric carboxyl (COO^-^) stretching vibrations characteristic of acetate ions, respectively. The bands at 1409–1444 cm^-1^ can also be attributed to asymmetric and symmetric stretching vibrations of the carboxyl group (COO^-^), indicating the presence of acetate ions. The absorption bands at 1346–1361, 660, and 643 cm⁻¹ can be attributed to OH bending vibrations, which may be related to water or hydroxyl groups present in the sample. The bands at 1028–1056 cm^-1^ are attributed to C–O stretching vibrations of the acetate ion, confirming the presence of acetate groups. The bands at 947, 858, 813, and 616 cm^-1^ are often associated with C–H bending vibrations of the acetate ion. The absorption bands at 485, 469, and 419 cm^-1^ are consistent with vibrations of metal-oxygen bonds, confirming the presence of calcium and magnesium^37,38^.

The absorption bands at 1240–1270 cm^-1^ in the compounds can be attributed to υ_c–o_ frequencies (acetate group). The strong absorption bands in the 1535–1630 cm^-1^ range in the compounds’ spectra can be assigned to a C$\:\frac{\dots\:\dots\:.}{}$O ligand. The shift around 50 cm^-1^ (C = O stretching vibration) toward a lower wavenumber may be due to the coordination of the carbonyl oxygen with the central calcium or magnesium atom^19,39^.

**Fig. 4:**
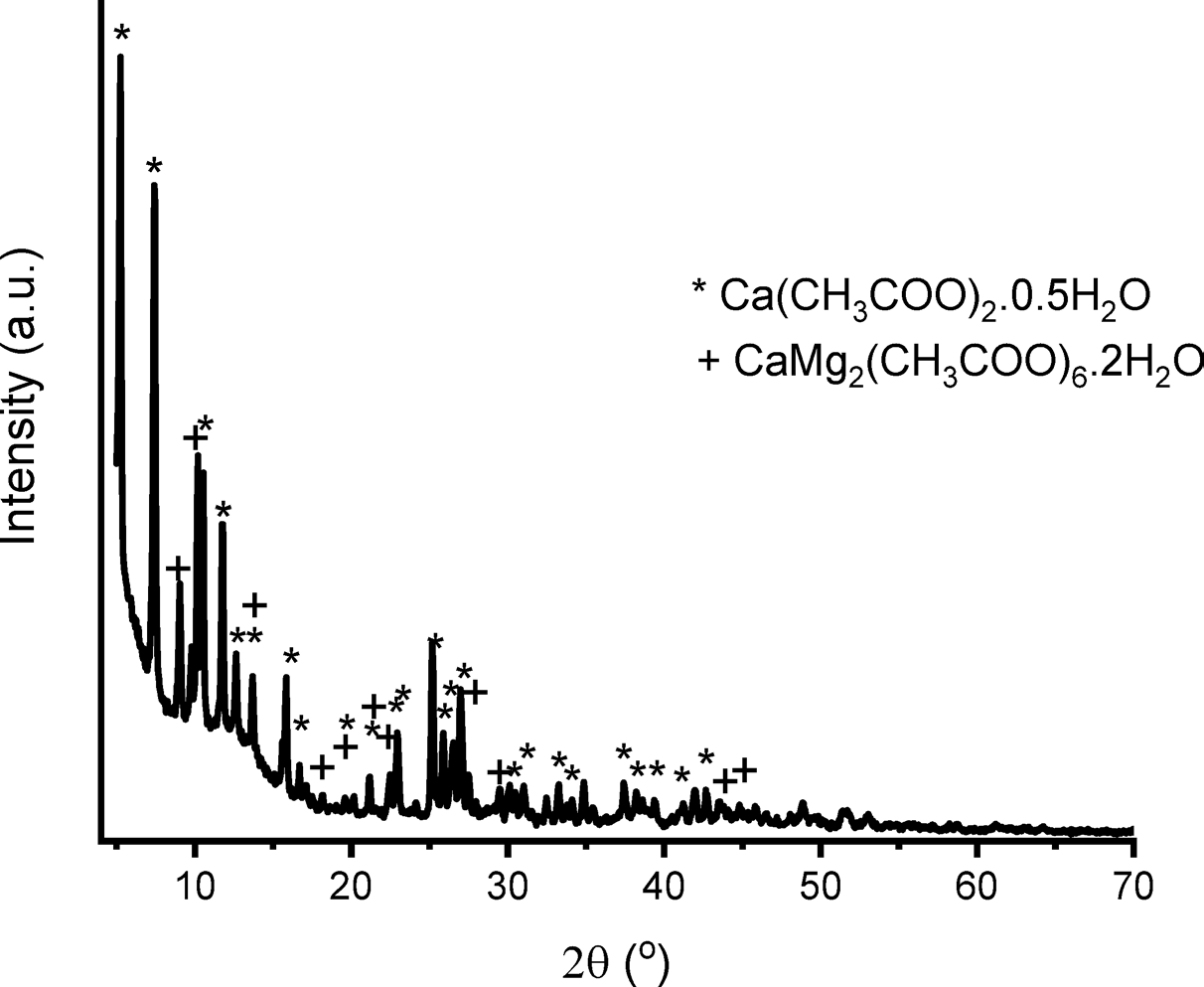
X-ray diffraction (XRD) pattern of calcium magnesium acetate de-icing agent.

X-ray diffraction analysis of CMA1 shown in Fig. 4 indicates that it consists of hydrated calcium acetate Ca(CH_3_COO)_2_.0.5H_2_O as the main component and dihydrated calcium magnesium acetate CaMg_2_(CH_3_COO)_6_.2H_2_O as a minor component^18,19,22,40–42^. This is consistent with the results of the elemental analysis using XRF as shown in Table 4.

### Adsorption evaluation

**Fig. 5:**
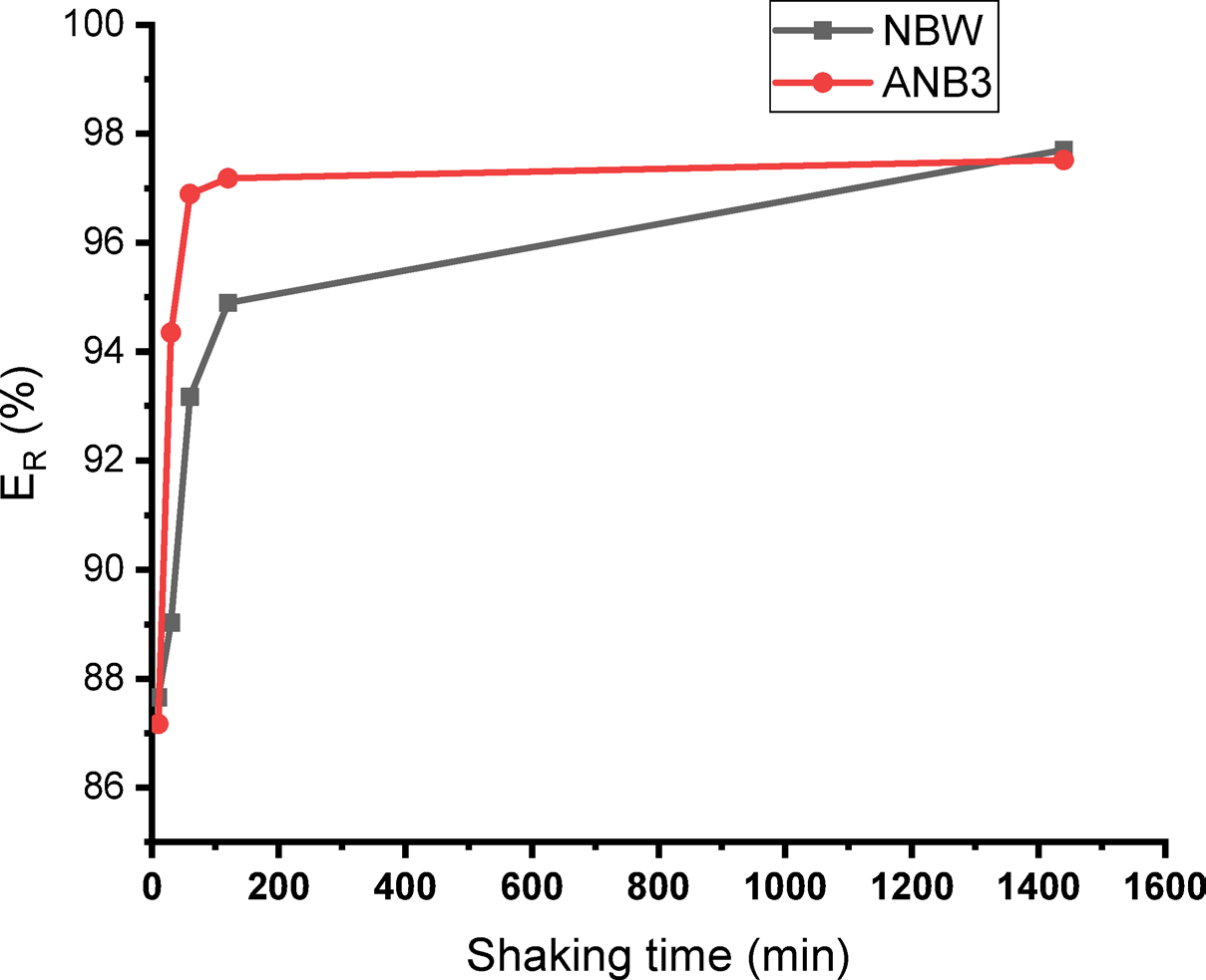
Effect of shaking time on the efficiency of MB dye removal (100 mg/L) using water-washed and acid-treated NB.

The effect of shaking time on the adsorption of methylene blue (MB) with both acid-treated and untreated NB was studied over a period ranging from 10 min to 24 h. The results in Fig. 5 indicate that equilibrium was nearly reached after 30 min of contact between the adsorbent and MB, after which the removal efficiency increased slowly. It was found that the activated NB (ANB3) demonstrated a better efficiency than the untreated one (NBW) within the first hour, achieving a separation efficiency greater than 93% for MB.

The kinetic model study results in Table 5 indicate that the pseudo-second-order model fits the adsorption processes of MB on both NBW and ANB3. The linear-fitting equations of kinetic models are shown in (supplementary information, S8).

**Fig. 6:**
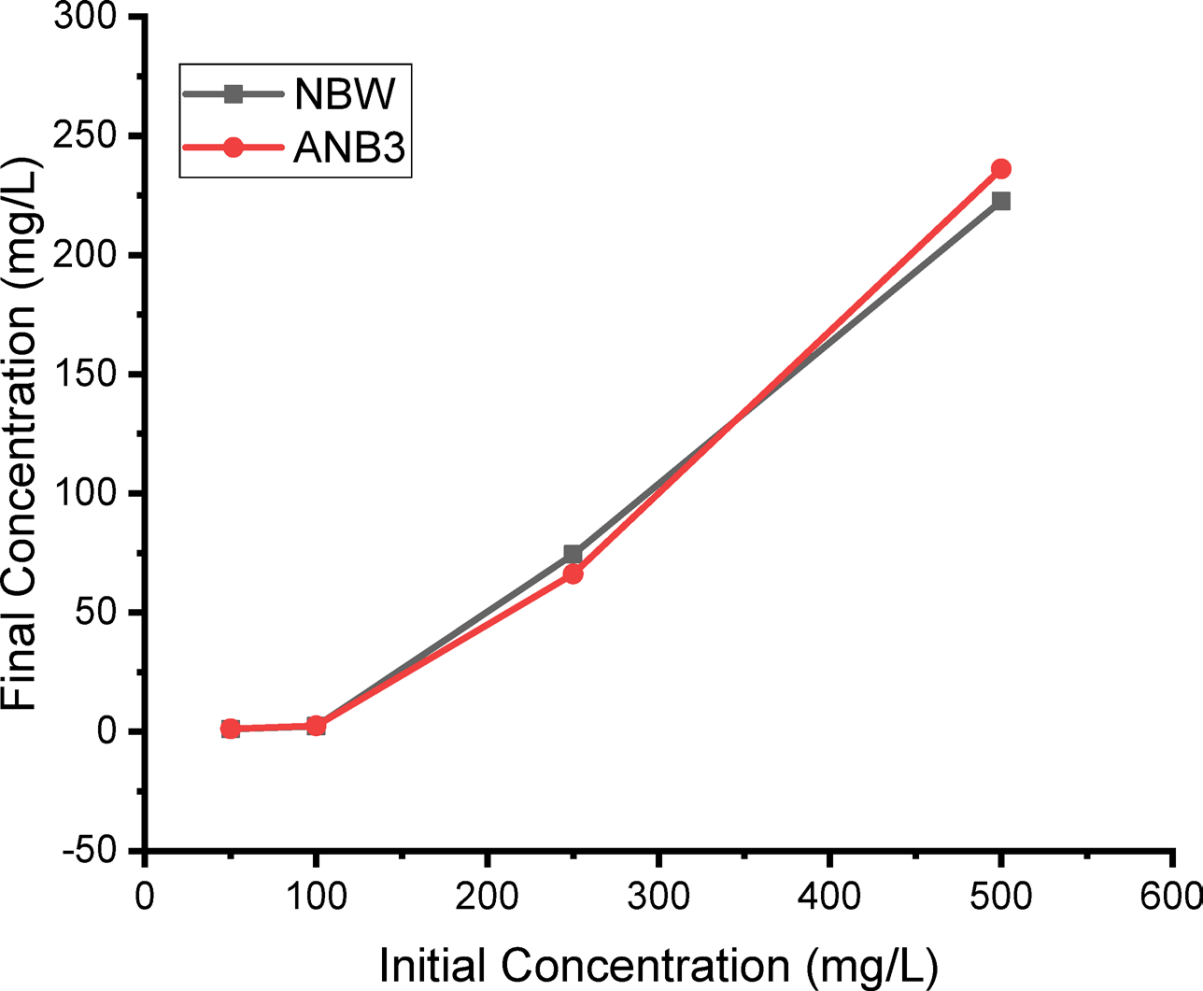
Effect of initial MB dye concentration on its final concentration after removal using water-washed and acid-treated NB.

The effect of different initial concentrations of MB, ranging from 50 to 500 mg/L, was studied, as shown in Fig. 6. The maximum removal efficiency was slightly over 97.5% at a MB initial concentration of 50 mg/L. Beyond this concentration, the removal efficiency decreased down to 52.8% at 500 mg/L. At low MB concentrations, adsorption sites in ANBs are abundant relative to dye molecules, resulting in high removal efficiency. Increasing dye concentration progressively saturates the available active sites in the activated Neptune balls, thereby reducing its removal efficiency. It is noted that untreated NB (NBW) performed slightly better than treated ones (ANB3), likely due to the deposited impurities.

**Table 4:** Quantitative XRF analysis of elements in CMA1.

Element (oxide form)	%
Na_2_O	0.83
Al_2_O3	0.052
CaO	25.656
Fe_2_O_3_	0.068
MgO	6.429
MnO	0.034
NiO	0.006
SrO	0.736
ZnO	0.01
SiO_2_	0.567
P_2_O_5_	0.025
SO_3_	0.396
K_2_O	0.11
Cl	0.068
I	0.014
LOI	65

From the adsorption isotherm models data (Table 6), the Freundlich model’s application indicated that the 1/n value was less than one, suggesting that the adsorption process was favorable within the concentration ranges studied. Both Langmuir and Freundlich models provided a good fit for the adsorption data, with NBW showing a slightly better fit with the Freundlich model and ANB3 with the Langmuir model. NBW had a slightly higher maximum adsorption capacity (q_max_) according to the Langmuir isotherm equation, while ANB3 exhibited a higher affinity towards MB as indicated by the higher K_L_ and K_F_ values, which is in accordance with the show slight better kinetics of equilibration of ANB3 compared with NBW. The linear correlations of the adsorption isotherm models are presented in the supplementary information (S9).

### Effect of initial pH on adsorption

**Fig. 7:**
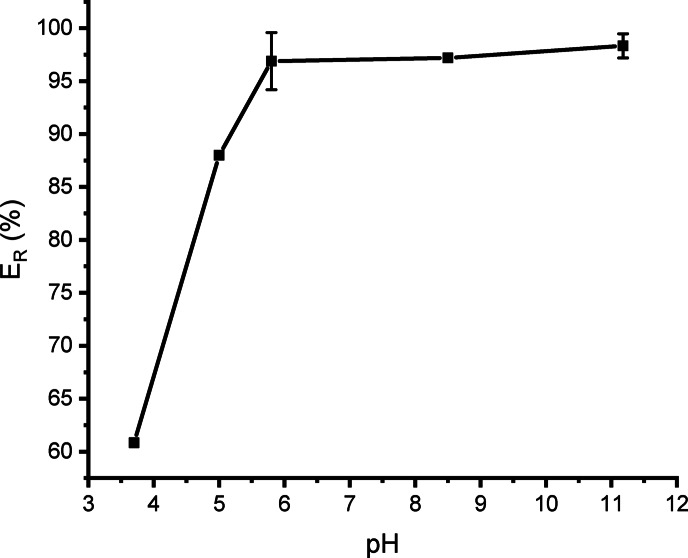
Effect of initial pH on the efficiency of MB dye removal (100 mg/L) using treated NB (ANB3). Error bars represent standard deviation from triplicate measurements under optimized experimental conditions.

The effect of initial pH on the removal efficiency of MB using the adsorbent ANB3 at a concentration of 2 g/L was studied for MB solutions with shaking for 60 min. Hydrogen ion concentrations were studied in the range of 3 to 12 for MB. Figure 7 shows that the removal efficiency of MB does not depend significantly on the pH from 6 to 12. At low pH, excess hydrogen ions compete with cationic MB molecules for adsorption sites, thereby reducing adsorption efficiency. At higher pH values, deprotonation of surface functional groups enhances electrostatic attraction toward MB.

Given that ANB3 has an adsorption capacity comparable to NBW, despite being utilized in producing extra economic materials, and due to its relatively strong and rapid binding with MB and its superior resistance to bacteria compared to NBW, it is considered the better option for application and further experiments.

### Adsorption mechanism

**Fig. 8:**
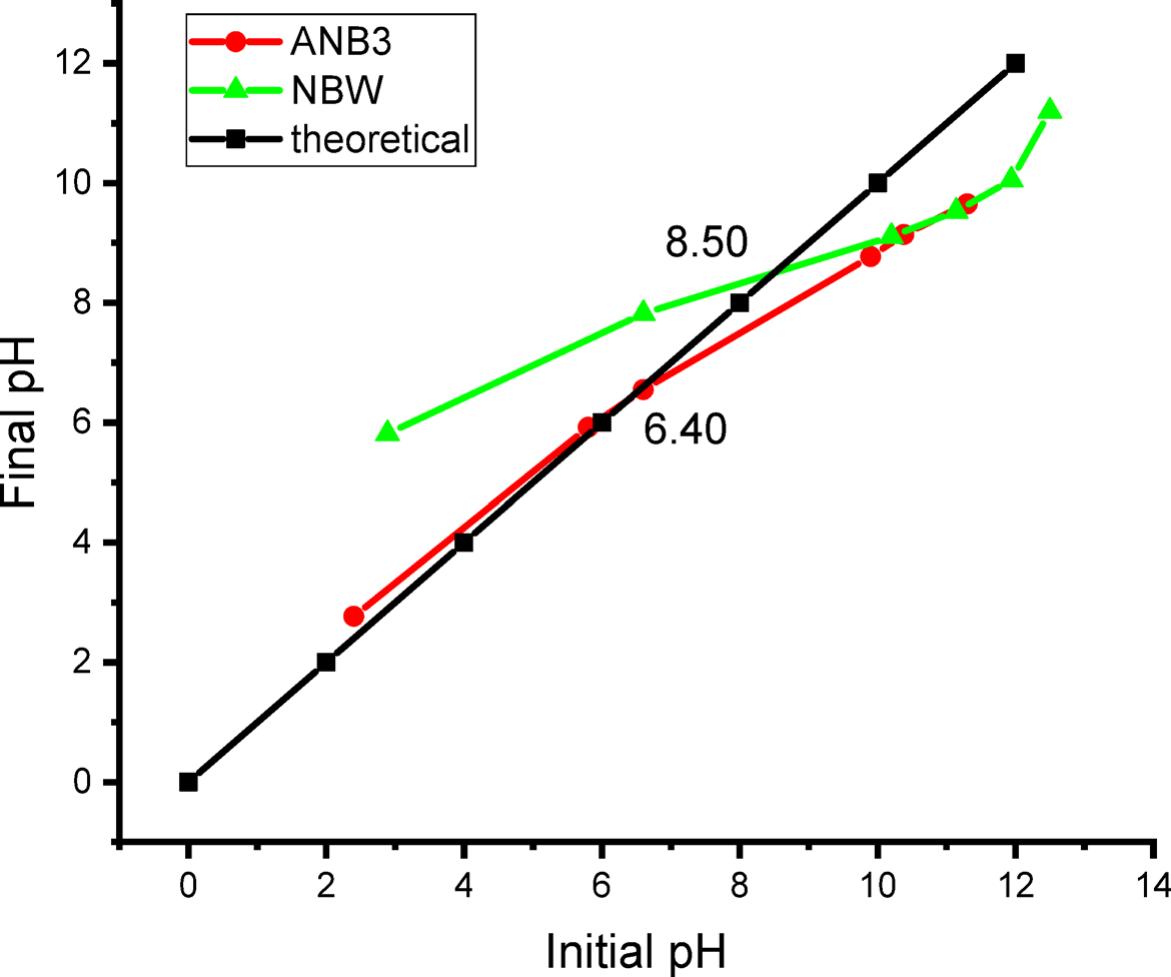
The pH_PZC_ of water-washed and acid-treated NB.

The pH_PZC_ values of NBW and ANB3 were determined to be 6.40 and 8.50, respectively (Fig. 8). The decrease in pH_PZC_ after acid activation suggests modification of the surface chemistry and the generation of additional acidic oxygen-containing functional groups. When the solution pH exceeds the pH_PZC_, deprotonation of hydroxyl and carboxyl groups results in a negatively charged surface, which enhances the adsorption of cationic methylene blue through electrostatic attraction. Conversely, at pH values below the pH_PZC_, protonation of the surface and competition between H⁺ ions and MB molecules reduce adsorption efficiency. The observed increase in MB removal at pH values higher than 5 is therefore consistent with the measured pH_PZC_ values.

**Fig. 9:**
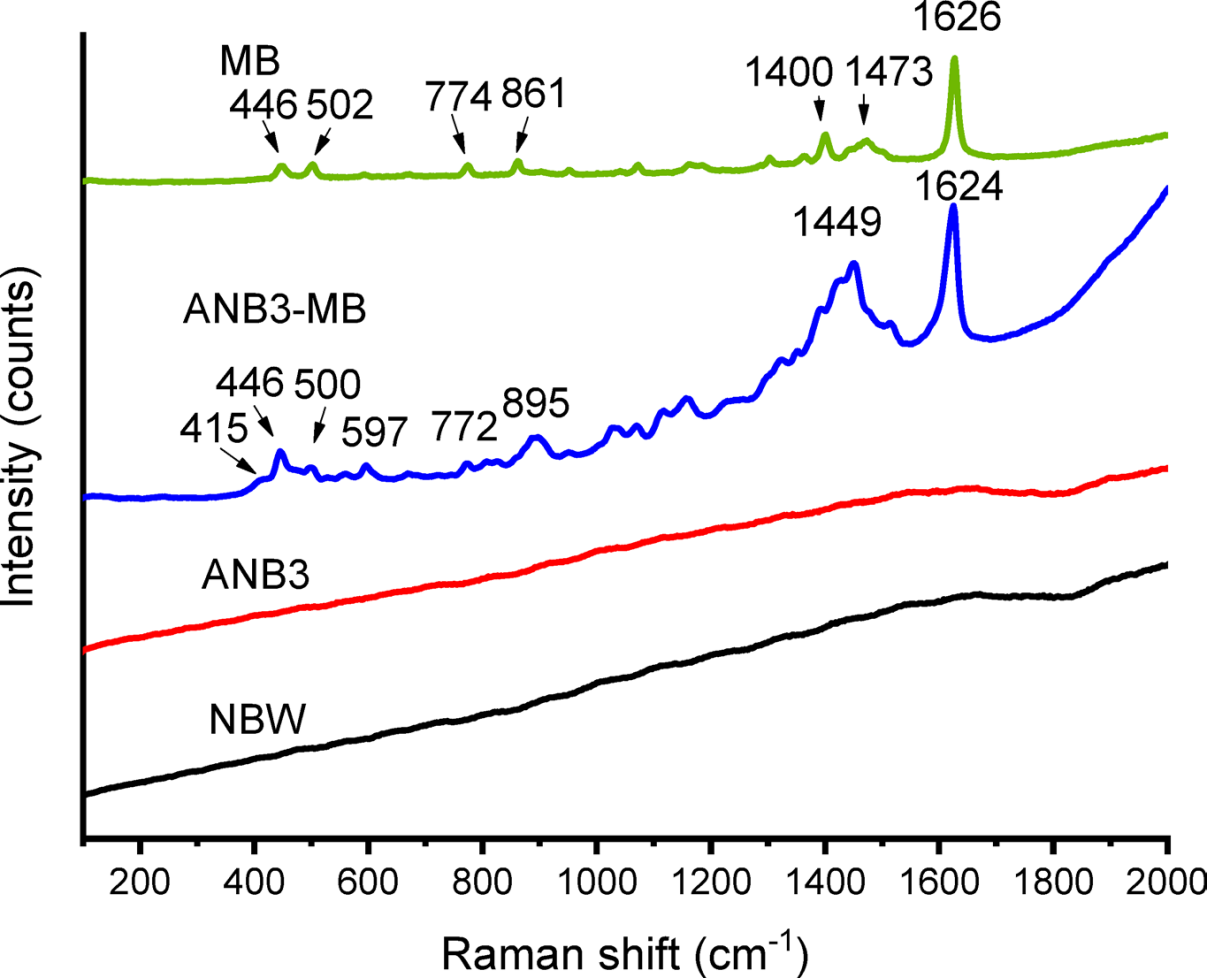
Raman shift spectrum of acid-treated NB (ANB3) before and after loading with MB dye.

Raman shift study of ANB3 before and after MB adsorption (Fig. 9) confirmed the loading of MB on it by identifying the characteristic Raman peaks of MB. Also, the optical microscopic image of the ANB3 loaded with MB (supplementary information, Fig. S10) shows clearly the blue coloration of the ANB3 fiber. The Raman spectrum of pure methylene blue exhibited characteristic bands at 446, 502, 774, 861, 1400, 1473, and 1626 cm^− 1^^43,44^. The Raman spectrum of ANB3-MB, showed broader and enhanced bands at approximately 415, 446, 500, 597,772, 895, 1449, and 1626 cm^− 1^. The persistence of the characteristic MB bands confirms successful adsorption of the dye onto the adsorbent surface.

According to previous Raman studies, the bands near 445–500 cm^− 1^ are particularly sensitive to the aggregation states of methylene blue and can be used to distinguish monomeric and dimeric species^44^. The observed broadening, noise increase and intensity enhancement observed in this region of the ANBB3 loaded with MB, together with the shifts of the bands from 861 to 895 cm^− 1^ and from 1400 to 1449 cm^− 1^, related to the symmetric and asymmetric stretch of C-N bond, suggest that adsorption on ANB3 modifies the aggregation state and local molecular environment of MB. The band at 1626 cm^− 1^, assigned to aromatic C = C stretching within the phenothiazine ring system^45^, remained essentially unchanged, indicating preservation of the molecular structure of MB after adsorption. Similar Raman shifts and band broadening have been reported for adsorbed MB and were attributed to dye–surface interactions involving electrostatic attraction and coordination of MB functional groups with adsorption sites on the adsorbent surface^43,46–48^.

Combined with the adsorption model, FTIR and pH_PZC_ results, the Raman data suggest that MB adsorption onto ANB3 proceeds firstly through electrostatic attraction between the cationic dye monolayer and negatively charged surface sites, followed by formation of multilayers of physically-adsorbed MB molecules. These multilayers are further stabilized by intermolecular interactions, including hydrogen bonding between dye molecules and hydroxyl/carboxyl groups present on the activated biomass surface. The observed spectral changes further indicate modification of the aggregation state of MB after adsorption.

**Table 5:** Kinetic parameters for the adsorption of 100 mg/L MB on NB.

Sample	Pseudo-first order				Pseudo-second order			
	q_e_ (mg g^− 1^) (exp.)	q_e_ (mg g^− 1^) (calculated)	K_1_ (min^− 1^)	*R* ^2^	q_e_ (mg g^− 1^) (exp.)	q_e_ (mg g^− 1^) (calculated)	K_2_ (g g^− 1^ min^− 1^)	*R* ^2^
NBW	48.85	5.60	0.0119	0.954	48.85	49.02	0.0075	0.999
ANB3	48.76	4.22	0.0299	0.85	48.76	48.78	0.0259	0.999

**Table 6:** Isotherm model parameters for MB adsorption on untreated and treated NB’s.

Adsorbent	q_max_ (mg/g)	*R*_L_	K_L_ (L/mg)	*R* ^2^
Langmuir isotherm parameters				
NBW	142.9	0.235 − 0.030	0.065	0.963
ANB3	135.1	0.194 − 0.023	0.083	0.986
Freundlich isotherm parameters				
NBW	0.279	29.289	0.917	
ANB3	0.282	28.874	0.925	

### Practical application of ANB3 for seawater treatment

The efficiency of MB removal was measured after raising its concentration to 50 mg/L in seawater samples collected from five different sites with varying compositions. This served as an example of the applicability of activated NB (ANB3) for removing cationic pollutants from mixed-contaminant waters. The samples were shaken with 1–2 g/L of activated NB for 60 min at room temperature and unmodified hydrogen ion concentration.

Table 7 shows the removal efficiency for MB added at percentages ranging from 57.8% to 87.5% with 1 g/L of activated NB, increasing to 60.5% to 89.4% with doubling the adsorbent amount. The study indicates that the separation process withstands changes in water nature, from pH to salinity or other content, confirming that the separation of cationic pollutants on modified NB is not affected by or interfering with the water contents and nature, providing practical evidence of the method’s viability as a simple and effective way to separate cationic pollutants, considering NB as locally available environmental waste. Figure S11 (supplementary information) shows optical illustration of the spiked seawater samples before and after separation.

Pearson correlation analysis (supplementary information, Table 9 and S12) between the adsorbent removal efficiencies towards MB and the saline water parameters; pH and TDS, was performed to examine their mutual effects. The results revealed a strong positive correlation between the MB removal efficiencies obtained at adsorbent doses of 1 and 2 g/L (*r* = 0.987, *p* = 0.002), indicating that both dosages exhibited highly similar adsorption behavior across the investigated seawater samples regardless the water parameters changes.

Moderate positive correlations were observed between pH and removal efficiencies (*r* = 0.550 and *r* = 0.629 for the doses 1 g/L and 2 g/L, respectively), whereas weak negative correlations were found between TDS and removal efficiency (*r* = − 0.195 and − 0.344 for the doses 1 g/L and 2 g/L, respectively). However, these correlations were not statistically significant (*p* > 0.05), likely due to the limited number of sampling sites (*n* = 5). Consequently, the results suggest that pH may positively influence methylene blue adsorption, while TDS exerts only a minor effect under the studied conditions. These results are in accordance with pH study and confirm that the activated seagrass is adopted with the saline water as its E_R_ values are independent on salinity.

**Table 7:** Efficiency of Purifying Seawater from Gouna, Suez - Red Sea - Egypt, Spiked with MB Using ANB3.

Site Name	pH	TDS (g/L)	Adsorbent Dose	E_*R*_ (%)	Adsorbent Dose	E_*R*_ (%)
Naval Base	8.01	34.2	1 g/L	60.4	2 g/L	60.5
Slaughterhouse Outfall	7.88	34.8	1 g/L	62.5	2 g/L	63.0
Near Fertilizers and Egypt Iran Co.	8.07	28.8	1 g/L	66.6	2 g/L	71.3
Near NIOF, Suez	8.08	31.6	1 g/L	57.8	2 g/L	61.2
Adabiya	8.14	32.0	1 g/L	87.5	2 g/L	89.4

Each seawater sample was analyzed once because each location represented an independent environmental sample.

**Table 8:** Pearson correlation testing of efficiency of purifying seawater from Gouna, Suez - Red Sea - Egypt, spiked with MB using ANB3.

Comparison parameter	pH	TDS (g/L)	E_*R*_ (%) Adsorbent Dose 1 g/L	E_*R*_ (%) Adsorbent Dose 2 g/L
pH	1	-0.66	0.55	0.63
TDS (g/L)	-0.66	1	-0.20	-0.34
ER (%) Adsorbent Dose 1 g/L	0.55	-0.20	1	0.99
ER (%) Adsorbent Dose 2 g/L	0.63	-0.34	0.99	1

### Application of CMA1 as an ice dissolving agent

Table 10 shows the results of dissolving 30 g of ice with the addition of 1–3 g of CMA1 compared to the same quantities of sodium chloride. The experiment is illustrated in Fig. S12 (supplementary materials). We observe that the dissolving power of both substances is equal throughout the experiment, giving an advantage to CMA1, as calcium magnesium acetate is an environmentally friendly solvent and is derived from environmental waste^18,41^.

**Table 9:** Comparison of MB adsorption parameters of ANB3 with different recent adsorbents.

Material	pH	Mono layer Capacity (mg/g)	E_*R*_ (%)	Equilibration time (min)	Adsorption isotherm	Adsorption kinetics	References
ANB3	6–12	135.1	97.5	30–60	Langmuir & Freundlich	2nd order	This work
NBW	–	142.9	97.7	1440	Freundlich	2nd order	This work
PO rhizomes	4–9	52.91	51.7–97.2%	30	Temkin	2nd order	^24^
PO leaves	2–12	175.44	91.5–99.9%	30	Dubinin–Radushkevich–Kaganer	2nd order	^25^
Graphene oxide	3–11	440.5-803.7	-	-	Langmuir-Freundlich	-	^49^
Activated carbon	9	384	99.9	60	Freundlich	2nd order	^50^
Activated starbones	-	891	98.5	240	Freundlich	2nd order	^51^
banana peels / MgO	7.6	76	96.45	30	Freundlich	2nd order	^52^

**Table 10:** Average dissolving rate of 30 g of ice with 1, 2 and 3 g of CMA or NaCl added at room temperature.

Time (min)	CMA	NaCl
20	20.0	23.5
40	54.8	55.3
60	97.8	97.2

## Conclusion

The acid treatment of NB with acetic acid successfully demonstrates the eco-friendly valorization of NB waste into valuable materials: an antibacterial adsorbent free of toxic elements (ANB3) and liquor rich with economic minerals, which upon drying turns into a green deicer of calcium magnesium acetates (CMA). ANB3 exhibited exceptional potential for environmental remediation, efficiently removing cationic dyes like MB from fresh or seawater with up to 89.4% removal efficiency. Its adsorption kinetics followed a pseudo-second-order model, with both Langmuir and Freundlich isotherms well-fitted to the data, indicating strong adsorption capacity. Notably, ANB3 maintained strong performance across varying pH from 6 to 12 and tolerant to salinity levels, emphasizing its suitability for practical applications. Additionally, its inherent antibacterial properties further support its suitability for water purification. CMA1 exhibits competitive deicing efficiency to NaCl, which recommend its beneficial usability regarding its eco-waste origin and green influence to environment.

The valorization of NB waste as functional adsorbents and green deicer, not only addresses waste recycling but also advances sustainable solutions for water treatment in addition to the forthcoming valuable substances from its extracts. These findings align with circular economy principles, offering scalable, eco-friendly green technologies that contribute to global sustainability goals. Future research could explore large-scale implementation and cost-effectiveness to maximize environmental and economic benefits.

## Supplementary Information

Below is the link to the electronic supplementary material.

Supplementary Material 1

## Data Availability

Authors shall make all data available at request.
