# Supplementary material for: Valorization of Posidonia oceanica balls: sustainable simultaneous synthesis of green de-icer and eco-friendly adsorbent for saline water treatment from methylene blue
Source: Sci Rep. 2026 Aug 13;16:25325. doi: 10.1038/s41598-026-66019-w (PMC13473686; doi:10.1038/s41598-026-66019-w)
Supplement: Supplementary file 1 [file 41598_2026_66019_MOESM1_ESM.docx]

Valorization of *Posidonia oceanica* Balls: Sustainable Simultaneous Synthesis of Green De-icer and Eco-friendly Adsorbent for Saline Water Treatment from Methylene Blue

Khaled S. Abou-El-Sherbini,^a;^ Randa R. Elmorsi,^b^

a. Department of Inorganic Chemistry, National Research Centre, 33 El Bohouth st. (former Eltahrir St.), P.O. 12622, Dokki, Giza, Egypt. Email: [ks.elsaid@nrc.sci.eg](mailto:ks.elsaid@nrc.sci.eg), [kh_sherbini@yahoo.com](mailto:kh_sherbini@yahoo.com).
b. National Institute of Oceanography and Fisheries (NIOF), Cairo, Egypt. Email: [rr.elmorsi@niof.sci.eg](mailto:rr.elmorsi@niof.sci.eg), [rrelmorsi@hotmail.com](mailto:rrelmorsi@hotmail.com).

**S1: Characterizing equipment**

Raman shift spectra were collected using a portable i-Raman Plus 532S laser Raman spectrometer equipped with a BAC151C Raman microscopic imaging system (B&W TEK, USA), using lenses ranging from 20-100X.

NC Soil Analyzer flash 2000 (location:- Italy ), Model : Thermo Scientific Flash2000, Serial No. :2015F0028, was used to determine the percentage of C, H, N, and S in the samples.

The point of zero charge (pH_PZC_) of the adsorbent was determined by adjusting the initial pH of 25 mL 0.1 M KCl solutions to a range of 2-12 using 0.01 M HCl or KOH. Then, 200 mg of the adsorbent was added to each solution. The suspensions were shaken in closed bottles for 24 h, and the final pH of the solutions was measured. The pH_PZC_ was then obtained from the intersection of the actual and theoretical graphs obtained by plotting the initial and final pH values.

UV/Vis spectra of MB solutions were recorded using a UV/VIS Peak Instrument – C7200 Spectrophotometer (PRC) at 663.8 nm. The concentration of MB was determined using a calibration curve prepared from standard MB solutions. The zeta potential of the aqueous sample suspensions was measured using a Zeta Sizer, ZS-Nano, from Malvern Instruments Ltd. (Malvern, UK). For zeta potential measurements, the sample was sonicated for 30-60 minutes before evaluation.

Colony Forming Unit (CFU) Test Method: *Staphylococcus aureus* (*S. aureus*) was used to evaluate the antibacterial activity of polymer sheets using the agar plating method. A suspension of *S. aureus* (0.5 McFarland standard) was prepared and incubated in Mueller Hinton broth. Then, 200 µL of the prepared bacterial suspension was added to a 96-well plate containing the test samples and a control sample (DMSO). The well plate was incubated at 37°C for 24 hours. Subsequently, 20 µL of the bacterial solution was cultured on the surface of dried nutrient agar plates, which were incubated at 37°C for 24 hours. The bacterial colonies on the plates were observed using a digital camera, and the number of colonies was counted. The antibacterial ratio was calculated as follows [1]:

Antibacterial ratio (%) = (Number of CFUs in control group - Number of CFUs in experimental group)/ (Number of CFUs in control group) ×100%

The antibacterial assay was performed in triplicate.

**S2**

**Figure S2 Neptune balls**

**S3**

**Figure S3: Photograph of raw calcium magnesium acetate (CMA1) de-icing agent.**

**S4**

**Figure S4: Photograph of raw calcium magnesium acetate (CMA2) de-icing agent.**

**S5**

**Figure S5 ANB3**

**S6: Adsorption studies**

Adsorption studies were conducted at room temperature (23 ± 1.0°C) and initial ambient pH of MB solutions was 5.8 unless stated otherwise. For studying the effect of time of shaking, batch experiments were carried out using 2.0 g L⁻¹ of adsorbent suspensions with initial concentration 100 mg L⁻¹ of methylene blue (prepared by dissolving methylene blue in deionized water) under shaking (180 rpm) for 10 - 1440 minutes.

For adsorption isotherm studies, batch experiments were carried out using 2.0 g L⁻¹ of adsorbent suspensions with initial concentrations ranging from 50 to 500 mg L⁻¹ of methylene blue (prepared by dissolving methylene blue in deionized water) under shaking (180 rpm) for 24 h. Although equilibrium was nearly achieved within 30–60 min, adsorption was monitored up to 24 h to confirm equilibrium stabilization and evaluate long-term adsorption behavior. The amount of dye adsorbed (mg g⁻¹) at equilibrium (q_e_) and at time t (qt) was calculated using the following mass balance expressions:

$q_{e}=\frac{(C_{0}-C_{e})}{m} V$	(1)

$q_{t}=\frac{(C_{0}-C_{t})}{m} V$	(2)

C_0_, C_e_ and C_t_ are the initial concentration, the equilibrium concentration and the concentration (mg L⁻¹) at time t, respectively of the dye. V and m are the volume of the solution (L) and the mass of the adsorbent (g), respectively.

To investigate the effect of initial pH on the adsorption behavior, 100 mg/L of MB was chosen as the initial concentration with an adsorbent dose of 2.0 g/L. The initial pH (2.0-12) of the MB solution was adjusted using 0.1 M NaOH and HCl solutions. The resulting suspension containing the adsorbent was shaken for 60 minutes and then centrifuged. The remaining concentrations of adsorbate in the solution were determined.

The loaded adsorbents, prepared by adding 20 mg of ANB3 to 10 mL of 100 mg L^-1^ MB at an initial pH of 5.8 and was shaken for 60 min, washed several times with DW, dried at 80 ºC for 24 h and stored for analysis.

**S7**

**Figure S7: Optical microscope images of NBW (a) and ANB3 (b).**

**S8: Kinetic Studies**

The rate constant of adsorption for the pseudo-first order kinetic model was calculated using Lagergren's pseudo-first order equation [2]:

*log (q_e_ – q_t_) = log q_e_ –* $\frac{k_{1}t}{2.303}$ (3)

where *k_1_* is the adsorption rate constant (min^–1^) and *q_e_* and *q_t_* are the MB adsorbed amounts (mg g^–1^) at equilibrium and at time t (min), respectively.

In the pseudo–second order kinetic model, the equation based on the equilibrium adsorption is expressed as in equation (4) [3]:

$\frac{t}{q_{t}}$ *=* $\frac{1}{k_{2}q_{e}^{2}}$ + $\frac{t}{q_{e}}$ (4)

where the second-order adsorption rate constant is represented by k_2_ (g mg^-1^ min^-1^).

**S9: Adsorption isotherms fitting**

According to the Langmuir isotherm model, monolayer sorption takes place on a homogenous surface during adsorption, with no contact between the molecules of the ANB3 surface [4]. The model postulates that there is no adsorbate transmigration in the surface plane and that adsorption energies on the surface are homogeneous. The following equation represents the Langmuir isotherm equation (5) in its linear version:

$\frac{C_{e}}{q_{e}}=\frac{1}{K_{L}Q}+\frac{1}{Q}C_{e}$ (5)

The equilibrium concentration of the adsorbate, the amount adsorbed at equilibrium, the monolayer adsorption capacity, and the Langmuir energy constant are represented by the values *C*_e_ (mg L^-1^), *q*_e_ (mg g^-1^), *Q* (mg g^-1^) and *K_L_* (L mg^-1^), respectively. Langmuir parameters, *Q* and *K_L_*, are derived from the slope and intercept of the isotherms. The separation factor, also known as the equilibrium parameter (*R_L_*), is a dimensionless constant that can be used to characterize the key characteristics of the Langmuir adsorption isotherm. It is defined by the following relationship:

$R_{L}=\frac{1}{1+C_{o}K_{L}}$ (6)

where *C_o_* represents the starting mg L^-1^ concentration of MB. The isothermal shape can be classified as irreversible (*R_L_* = 0), advantageous (0 < *R_L_* < 1), linear (*R_L_* = 1), or unfavorable (*R_L_* > 1) based on the *R_L_* value.

The sorption equation was also described the Freundlich isotherm [5]. The following equation represents this somewhat adequate empirical isotherm, which can be applied to non-ideal sorption involving heterogeneous surface energy systems:

*ln q_e_ = ln K_F_ +1/n ln C_e_*	(7)

where 1/*n* is the adsorption intensity and *K*_F_ (mg/g (L mg^-1^)^1/n^) is an approximate measure of the adsorption capacity. While the size of the exponent 1/*n* indicates the favorability of adsorption, the *K*_F_ value generally increases with the adsorbent's adsorption capability for a given adsorbate.

**S10**

Figure S10: Photomicrograph demonstrating the efficiency of MB dye removal (initial concentration 50 mg/L).

**S11**

Fig. S11 Separation efficiency of methylene blue dye at an initial concentration of 50 mg L⁻¹ from natural water samples from (1) a marine base, (2) the slaughterhouse outfall, (3) in front of the fertilizer plant and the Egypt-Iran companies, (4) in front of the National Institute of Oceanography and Fisheries in Suez, and (5) Al-Adabiyah, to which additional doses of 1.0 and 2.0 g L⁻¹ were added to 5 mL of the water sample for 60 minutes at ambient pH and room temperature.

Table S11 Raw Pearson Correlation Testing of Efficiency of Purifying Seawater from Gouna, Suez - Red Sea - Egypt, Spiked with MB Using ANB3

| **Correlations** | | | | | | | |
| --- | --- | --- | --- | --- | --- | --- | --- |
|  | | TDS | dose1 | Er1 | dose2 | Er2 | pH |
| TDS | Pearson Correlation | 1 | .^a^ | -.195 | .^a^ | -.344 | -.660 |
|  | Sig. (2-tailed) |  | . | .754 | . | .570 | .225 |
|  | N | 5 | 5 | 5 | 5 | 5 | 5 |
| dose1 | Pearson Correlation | .^a^ | .^a^ | .^a^ | .^a^ | .^a^ | .^a^ |
|  | Sig. (2-tailed) | . |  | . | . | . | . |
|  | N | 5 | 5 | 5 | 5 | 5 | 5 |
| Er1 | Pearson Correlation | -.195 | .^a^ | 1 | .^a^ | .987^**^ | .550 |
|  | Sig. (2-tailed) | .754 | . |  | . | .002 | .337 |
|  | N | 5 | 5 | 5 | 5 | 5 | 5 |
| dose2 | Pearson Correlation | .^a^ | .^a^ | .^a^ | .^a^ | .^a^ | .^a^ |
|  | Sig. (2-tailed) | . | . | . |  | . | . |
|  | N | 5 | 5 | 5 | 5 | 5 | 5 |
| Er2 | Pearson Correlation | -.344 | .^a^ | .987^**^ | .^a^ | 1 | .629 |
|  | Sig. (2-tailed) | .570 | . | .002 | . |  | .256 |
|  | N | 5 | 5 | 5 | 5 | 5 | 5 |
| pH | Pearson Correlation | -.660 | .^a^ | .550 | .^a^ | .629 | 1 |
|  | Sig. (2-tailed) | .225 | . | .337 | . | .256 |  |
|  | N | 5 | 5 | 5 | 5 | 5 | 5 |
| a. Cannot be computed because at least one of the variables is constant. | | | | | | | |
| **. Correlation is significant at the 0.01 level (2-tailed). | | | | | | | |

**S13**

Figure S13 Photograph (and) Ice melting experiment (30g) using 1, 2, or 3 grams of CMA1 compared to the same quantity of NaCl.

References of supplementary information

1.	Xiang, Y. *et al.* Infection-prevention on Ti implants by controlled drug release from folic acid/ZnO quantum dots sealed titania nanotubes. *Materials Science and Engineering C* **85**, 214–224 (2018).

2.	Lagergren, S. Zur Theorie der sogenannten Adsorption gelöster Stoffe. *Handlingar* **24**, 1 (1898).

3.	Ho, Y. S. S. & McKay, G. Pseudo-second order model for sorption processes. *Process Biochemistry* **34**, 451–465 (1999).

4.	Langmuir, I. The adsorption of gases on plane surfaces of glass, mica and platinum. *J. Am. Chem. Soc.* **40**, 1361–1403 (1918).

5.	Freundlich, H. M. F. Uber die adsorption in losungen, Z. *Phys. Chem.* **57A**, 385–470 (1906).
